# On the Inverse Correlation of Protein and Oil: Examining the Effects of Altered Central Carbon Metabolism on Seed Composition Using Soybean Fast Neutron Mutants

**DOI:** 10.3390/metabo10010018

**Published:** 2019-12-28

**Authors:** Shrikaar Kambhampati, Jose A. Aznar-Moreno, Cooper Hostetler, Tara Caso, Sally R. Bailey, Allen H. Hubbard, Timothy P. Durrett, Doug K. Allen

**Affiliations:** 1 Donald Danforth Plant Science Center, St. Louis, MO 63132, USA; skambhampati@danforthcenter.org (S.K.); cooperhostetler@gmail.com (C.H.); tsc152@humboldt.edu (T.C.); ahubbard@danforthcenter.org (A.H.H.); 2 Department of Biochemistry and Molecular Biophysics, Kansas State University, Manhattan, KS 66506, USA; ja.aznar@upm.es; 3 United States Department of Agriculture, Agricultural Research Service, St. Louis, MO 63132, USA; sally.bailey@usda.gov

**Keywords:** soybeans, fast neutron mutants, protein, oil, raffinose family oligosaccharides, correlations, linear models, central carbon metabolism, seed development

## Abstract

Protein and oil levels measured at maturity are inversely correlated across soybean lines; however, carbon is in limited supply during maturation resulting in tradeoffs for the production of other reserves including oligosaccharides. During the late stages of seed development, the allocation of carbon for storage reserves changes. Lipid and protein levels decline while concentrations of indigestible raffinose family oligosaccharides (RFOs) increase, leading to a decreased crop value. Since the maternal source of carbon is diminished during seed maturation stages of development, carbon supplied to RFO synthesis likely comes from an internal, turned-over source and may contribute to the reduction in protein and lipid content in mature seeds. In this study, fast neutron (FN) mutagenized soybean populations with deletions in central carbon metabolic genes were examined for trends in oil, protein, sugar, and RFO accumulation leading to an altered final composition. Two lines with concurrent increases in oil and protein, by combined 10%, were identified. A delayed switch in carbon allocation towards RFO biosynthesis resulted in extended lipid accumulation and without compromising protein. Strategies for future soybean improvement using FN resources are described.

## 1. Introduction

Soybean (*Glycine max*) is a versatile and important crop with many uses. In 2017, 80 million acres of soybeans were planted in the United States (United States Department of Agriculture (USDA), National Agricultural Statistics Service (NASS)). The value of soybean is mostly due to high protein content with balanced amino acid composition that is used to produce feed for livestock and high seed oil content that is a primary source of vegetable oil and the feed stock for biofuel production. Soybean protein represents approximately 35–40% of the total biomass in soybeans and is the component of greatest value in soybean meal. Depending on the cultivar and planting zone [1,2,3], an additional 3–12% of biomass is present as sugars and oligosaccharides [4]. Sugars, such as sucrose, represent a metabolizable energy source in animal feed, but raffinose and stachyose that are members of the raffinose family oligosaccharides (RFOs) are indigestible and undesirable for livestock production [5,6]. RFOs have hypothesized roles in seed desiccation tolerance [7,8,9], stability of liposomes during dehydration, and seed germination [10,11,12], although efforts in breeding and genetic engineering have demonstrated that reduced levels do not impact seed viability [13,14,15]. The RFO and sucrose levels are the result of central carbon metabolism and could adversely impact the levels of oil and protein, which are known to be inversely correlated in mature seeds [16,17,18,19,20,21].

**Figure 1:**
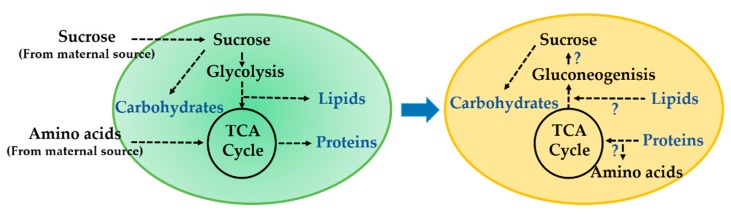
Drawing showing hypothetical shift in carbon partitioning from seed filling to maturation phase. During seed filling (R5–R7), central carbon (C) and nitrogen (N) sources (i.e., sucrose and amino acids) from maternal tissue are distributed to lipids, carbohydrates, and proteins through glycolysis, tricarboxylic acid (TCA) cycle, and amino acid metabolic pathways. As the seed transitions to maturation phase (R7 to R8), some accumulated proteins are degraded by proteolysis, while lipids are degraded via β-oxidation and potentially used for raffinose family oligosaccharide (RFO) biosynthesis through gluconeogenesis.

Protein and lipid accumulation over the course of seed development is dependent on the supply of amino acids and sugars from the maternal sources (i.e., organic carbon assimilated in the leaf) as well as the metabolism within the developing seed (i.e., cotyledons) [22,23,24,25]. The levels of intermediates of central carbon (C) and nitrogen (N) metabolism that are precursors for storage reserve production, vary depending on the stage of reproductive development and may indicate temporal changes in metabolism [26,27,28]. During maturation, 10–15% of lipids are degraded [28,29,30], coinciding with RFO accumulation, and occurring at the time when there are little to no exogenous resources supplied by the maternal plant. The expression of genes involved in gluconeogenesis and glyoxylate cycle suggest carbon remobilization from lipid may occur. Along with existing sucrose present late in development, carbon from lipid could contribute to central metabolism and enable RFO production during the maturation phase. Protein may also contribute carbon through turnover induced by proteases expressed late in development [28,31]. Figure 1 summarizes the hypothetical shift in metabolism from seed fill to maturity.

The relationship between the accumulation of protein and oil has been examined. Through in vivo embryo culture, changes in the supply of C and N sources resulted in inverse correlations between the two biomass components [22,32]. Based on the changes to maternal supply and seed metabolism that occur over the course of seed development, we hypothesized that in late seed development, carbon derived from the turnover of lipids and proteins contributes to the synthesis of RFOs. This concept was tested by inspecting correlations between biomass components in developing soybean seeds. Fast neutron (FN) lines, which had differences in genes involved in C and N partitioning, were evaluated.

Fast neutron (FN) mutagenesis-based forward genetic screens have been successful at identifying the genetic basis of valuable phenotypes in soybean [33,34,35,36]. Advances in the use of whole genome sequencing and comparative genome hybridization (CGH) techniques for mapping the deletions in mutant populations [37,38,39] also provide a means to identify novel mutations in genomic studies [40,41] that can have applications in plant breeding programs for improved agronomic traits. Studying the role of altered C and N partitioning as a function of development may provide clues to overcome the inverse correlation between oil and protein and to develop soybean lines with improved seed composition.

## 2. Materials and Methods

### 2.1. Plant Material and Growth Conditions

Soybean (*Glycine max*) seeds selected for gene mutations in central carbon metabolism were obtained from two fast neutron (FN) mutagenized populations developed at the University of Missouri (MO) and the University of Minnesota (MN) with the USDA (Table 1). Seeds were grown in greenhouses at Kansas State University (KSU) in 2018 for initial lipid and protein evaluation with fatty acid methyl ester (FAME) (described in Section 2.3) and NIR analysis, respectively. One-gallon pots were filled with 4:2:1 mixture of Metromix Professional Growing Mix/Vermiculite/Perlite supplemented with Osmocote 14-14-14 slow release fertilizer. Plants were watered three times per week and grown with day/night temperatures ranging from 25 °C to 27 °C/21 °C to 23 °C and humidity maintained at 45% or greater. Supplemental light (400–1000 W m^−2^) was used to maintain a 14 h day/10 h night photoperiod. Select FN lines were replanted in 2019, at the Donald Danforth Plant Science Center. Two seeds from each mutant line were planted in individual one-gallon pots containing Fafard 4M soil and grown in greenhouses. Temperature, humidity, and light conditions were maintained similar to the conditions at KSU. Plants were watered daily and fertilized with Jack’s 15-16-17 fertilizer (JR Peters, Allentown, PA, USA) three times per week. Pods were harvested from each plant at R5, R6, R7, R7.5, and R8 stages of reproductive development [42,43] and placed directly on ice. Seeds were removed from the pods, seed coats were excised, and the cotyledons were flash frozen in liquid nitrogen. Samples were stored at −80 °C until further use.

### 2.2. Extraction of Lipids, Protein, and Soluble Sugars

Following the method described in Ma et al. [44] with limited modifications, lipids, protein, and soluble sugars were obtained from the cotyledons (analyzed as single seeds). Stored samples were removed from −80 °C and placed in liquid nitrogen, two metal beads were then added to each tube, and the samples were pulverized using a ball mill at 30 Hz for 25 s for three rounds or until completely powdered. Pulverized samples were lyophilized for ~48 h at 2.4 Pa and −48 °C. After lyophilization, the metal beads were removed, and the dry weight of the sample was obtained. Samples were collected in triplicate from two individual plants to obtain a total of six replicates used for lipid and soluble sugar extraction and another six for protein hydrolysis.

To extract and quantify the lipids and soluble sugars, 1 mL 7:3 methanol/chloroform (−20 °C), a triheptadecanoin standard, a tripentadecanoin standard, and a norvaline/ribitol mixed standard were added to each sample. Samples were vortexed and mixed at 4 °C for ~2 h with all subsequent steps occurring at 4 °C. A 500 µL portion of ddH_2_O was added to each sample prior to vortexing, then centrifugation at 14,000 rpm for 10 min, to generate three distinct phases. The upper aqueous phase, containing water-soluble metabolites (sugars and free amino acids), was transferred to a 1.5 mL tube. The samples containing soluble sugars were dried in a speed vacuum centrifuge (Labconco^®^, Kansas City, MO, USA) and stored at −80 °C for LC/MS analysis. A 200 µL portion of 100% methanol was added to the remaining ~300 µL organic phase (containing lipids) and protein layers. The samples were vortexed and centrifuged at 14,000 rpm for 10 min, resulting in a pellet that contained insoluble proteins and carbohydrates along with an upper layer of chloroform/methanol. This upper layer was then transferred to an 8 mL glass vial and dried in a speed vacuum centrifuge to be processed into fatty acid methyl esters (FAMEs).

Protein quantification was performed by hydrolyzing the peptide bonds and quantifying the resulting individual amino acids as described in Kambhampati et al. [45]. Protein hydrolysis was performed in 100 µL of 4 M methanesulfonic acid with 0.2% tryptamine spiked with 20 µL of 1 mM ^13^C ^15^N-labeled amino acid standard mix. Samples were incubated at 110 °C for ~22 h followed by neutralization with 100 µL of 4 M NaOH. Samples were then completely dried down and 1 mL 50% MeOH was added to resuspend hydrolyzed amino acids prior to filtering with a 0.8 µM PES membrane centrifuge filter. Chromatography and mass spectrometry conditions for separation and detection of amino acids were identical to the method described in Kambhampati et al. [45].

### 2.3. FAME Analysis for the Quantification of Lipids

Analysis of lipid content was carried out according to an adapted version of the method described in Allen and Young [22]. In brief, freshly prepared 5% sulfuric acid:methanol (*v*/*v*) was added to the dried product along with 25 µL 0.2% butylated hydroxytoluene (BHT) in methanol to prevent oxidation before being vortexed and heated at 110 °C for 2.5–3 h, vortexing hourly. After cooling to room temperature, 0.9% NaCl (*w*/*v*) was added to each sample to quench the reaction. The FAMEs were then extracted using hexane and quantified by gas chromatography–flame ionization detection (GC-FID) using a DB23 column (30 m, 0.25 mm i.d., 0.25 μm film; J&W Scientific, Folsom, CA, USA). The GC was operated in a split mode (50:1). The flame ionization detector was operated with a temperature of 250 °C with an oven program that ramped from 180 °C to 260 °C at a rate of 20 °C min^−1^ followed by a hold time of 7 min. Comparisons of peak areas to the two internal standards were used for quantification.

### 2.4. Quantification of Sugars using HILIC-MS/MS

The aqueous phase samples containing water-soluble metabolites were separated for sugars and analyzed using a Shimadzu HPLC system (UFLCXR, Columbia, MD, USA) connected to an AB Sciex 6500 triple quadrupole/trap MS instrument equipped with Turbo V™ electrospray ionization (ESI) source. Negative ion mode was used for ionization of sugars. A 3 µL sample was injected on the Infinity Lab Poroshell 120 Z-HILIC column (2.7 µm, 100 × 2.1 mm; Agilent Technologies, Santa Clara, CA, USA) and the metabolites were eluted with an increasing gradient of acetonitrile/10 mM ammonium formate (90:10 *v*/*v* ACN:H_2_O) (A) and 10 mM ammonium formate in water (B) with pH adjusted to 6.9 for both buffers. Both the buffers were spiked with 5 µM medronic acid to improve compound retention on the column. The flow rate used was 0.20 mL/min. Sugars were separated using a binary gradient of 100–70% B over 5 min and then to 30% B over the next 7 min followed by a hold at 30% B for 1 min. The gradient was then returned to 100% B over the next minute before re-equilibrating the column for six minutes. Eluted compounds separated by the HPLC were introduced to the mass spectrometer by an electrospray ionization (ESI) source with the following conditions: ion spray voltage, 4.5 kV (ESI-); ion source temperature, 550 °C; source gas 1, 45 psi; source gas 2, 40 psi; curtain gas, 35 psi; and entrance potential, 10. Ions were detected and monitored using a targeted multiple reaction monitoring (MRM) approach with the parameters listed in Table S1 (Supplementary Materials). The value for entrance potential was default (−10) for all analytes. Other parameters for MRMs were optimized using direct injections of individual sugar standards. Data were analyzed using the quantitation wizard available in Analyst (v. 1.6.2) software (AB SCIEX, Concord, ON, Canada). Metabolite concentrations and recoveries were calculated based on a calibration curve and ribitol as an internal standard, respectively.

### 2.5. Linear Modeling and Statistical Analyses

Correlations between bulk proteins, lipids, and oil measurements were calculated at the developmental time points R5, R6, R7, R7.5, and R8. Multiple measurements taken from a single plant made it possible to observe the impact of developmental time on correlations between pairs of compounds and to determine if the levels at one time point are predictive of levels at the other time points. Linear modeling, implemented in R programing language, was then used to determine if the relationship between a pair of compounds changed as a function of time during at least one developmental stage (*p*-value of interaction term <0.05). For each pair of metabolites (A,B), models of the form A ~ time point + B*time point and B ~ time point + A*time point were calculated.

## 3. Results

### 3.1. Identification of FN Lines with Alterations in Central Carbon Metabolic Genes

A total of 25 lines from two core collections of soybean fast neutron mutant population were selected. The first collection consisted of 8 lines from a mutant population developed at the University of Missouri, Columbia from the “Williams 82” background and the second consisted of 17 lines from a mutant population developed at the University of Minnesota and USDA using the background “M92-220” [37]. A list of the FN descriptors for the lines, 50-seed weight at maturity, and the selection criteria for central carbon metabolic gene deletions or oil phenotype are presented in Table 1. In order to assess the oil content and stability of loci leading to a high oil phenotype within these populations, total oil content at maturity was measured from four individual seeds selected from four different plants for each of the selected lines. Three lines from the Williams 82 mutagenized population and eight from the M92-220 mutagenized population showed a high oil phenotype (Figure S1, Supplementary Materials).

**Figure 2:**
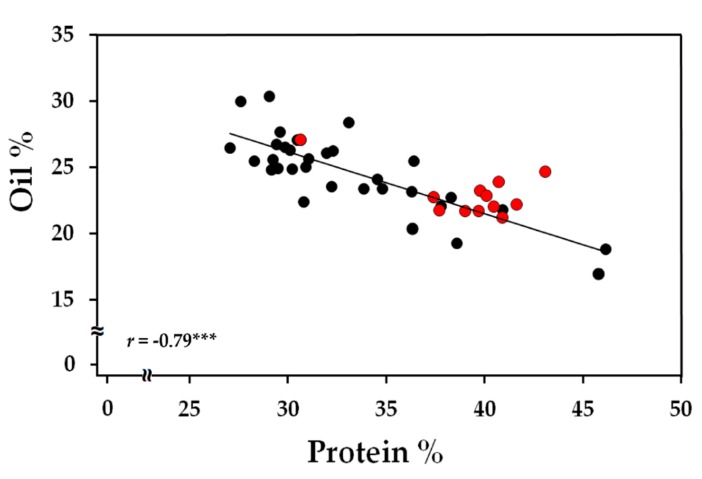
Correlation plot for oil and protein content represented as % biomass at maturity. A total of 11 FN lines with four biological replicates were used in the analysis. Red markers represent the three lines, FN300660, FN300012, and FN301952, from the Williams 82 mutagenized population. *r* represents the Pearson’s correlation coefficient and *** indicates a significance of *p* < 0.001.

The protein content in the selected eleven lines along with the two wild types, Williams 82 and M92-220 (Table S2, Supplementary Materials), was measured to assess the relationship between oil and protein. An overall negative correlation was observed between the two biomass components with a Pearson’s correlation coefficient, *r* = −0.79 and *p* < 0.0001 (Figure 2). However, in three FN lines under Williams 82 background (FN300660, FN300012, and FN301952), the high oil content did not result in a significant penalty in the protein level. The seeds obtained from these lines were replanted in 2019 for biomass component description over the course of seed development in the subsequent generation.

### 3.2. FN300012 and FN301952 Accumulated High Oil without a Decline in Their Total Protein Content

**Figure 3:**
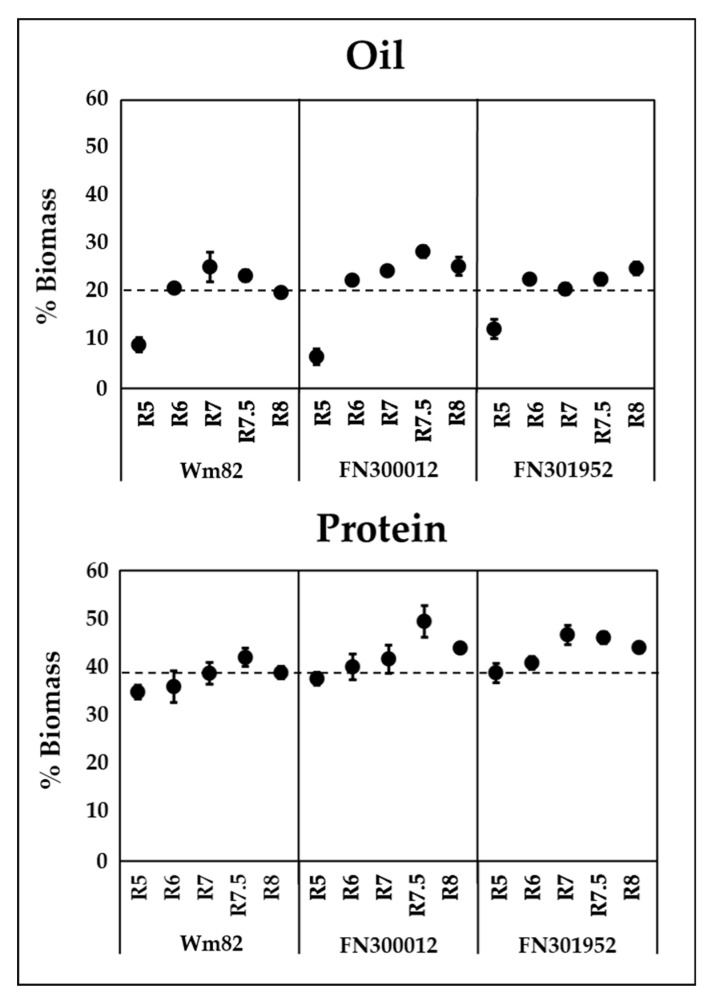
Oil (top panel) and protein (bottom panel) content in cotyledons over the course of development in two FN lines FN300012 and FN301952 compared to the wild-type Williams 82 (Wm82). Dotted lines represent the oil and protein quantities of the wild type at maturity for comparison. Error bars are the standard error of the mean (SEM), *n* = 6. Absolute values are presented in Table S3.

Total oil and protein contents in cotyledons were quantified in all three lines and the wild-type Williams 82 over the course of development from R5 to R8. Reproductive developmental stages were defined based on the seed weights (Table S3, Supplementary Materials) and were consistent with previous descriptions [42,43]. FN300660 displayed large variation in protein and oil accumulation based on single-seed analysis, likely due to segregation of causal genes for the metabolic phenotype and hence was not pursued further. The accumulation of oil over the course of development has been previously reported for Williams 82 [28]. A similar trend was observed in our study, where the content increased up to R7 and declined during the maturation phase (R7 and R8). This decline was delayed in line FN300012 and no decrease in oil content was observed in FN301952 (Figure 3). Protein accumulation in Williams 82 continued to R7.5 and declined towards the end. No difference in the trends of accumulation or decline were observed for protein between the two FN lines and Williams 82; however, the quantity of protein accumulated reached as high as 49.5 ± 3% in FN300012 at R7.5 and 46.2 ± 1% in FN301952 at R7, whereas the highest protein content in Williams 82 reached only 42.1 ± 2% at R7.5. The overall protein content at maturity was also elevated in both the mutant lines (44 ± 1%) compared to the wild type (38.9 ± 1%). The increased protein content in mutants was expected to be accompanied by reduced oil levels, based on the inverse correlation between protein and oil [16,17,18,19,20,21], yet an overall increase in oil content at maturity was also observed for both the mutant lines (25.5 ± 2%, 25 ± 1.3% for FN300012 and FN301952, respectively) relative to the wild type (20 ± 0.7%).

### 3.3. No Decrease in RFOs Was Observed in the Final Composition of Selected Lines

**Figure 4:**
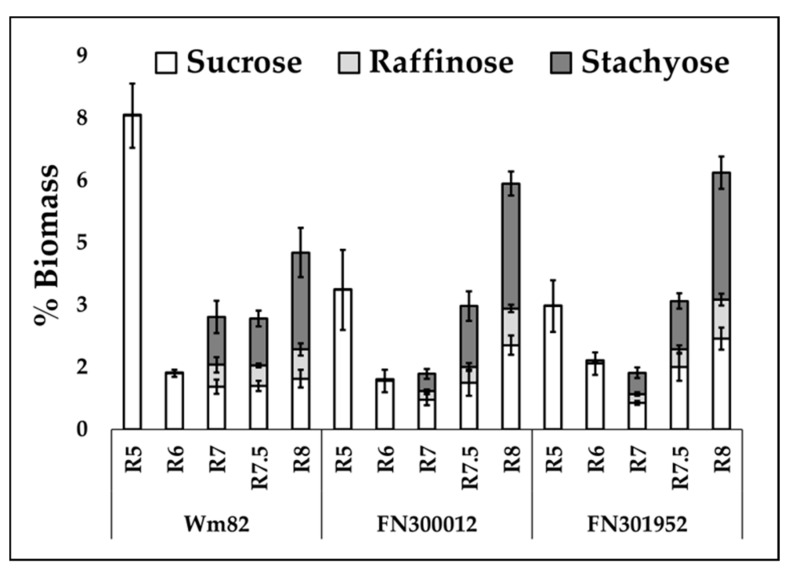
Sucrose, raffinose, and stachyose content in the two lines, FN300012 and FN301952, compared to Williams 82 over the course of development. Error bars represent standard error of the mean (*n* = 6). Absolute values are presented in Table S3.

RFOs constitute 3–12% of biomass at maturity [1,2,3] and are a significant carbon sink during the maturation phase. Since the stages corresponding to the decline in oil coincide with the accumulation of RFOs in Williams 82 (Figure 4), we hypothesized that there was a change in resource allocation to accumulate raffinose and stachyose (the most abundant RFOs) along with sucrose in the mutant lines. The total raffinose and stachyose content was found to be ~2.5-fold lower in both the mutant lines (FN300012, FN301952) compared to Williams 82 at R7 (Figure 4), indicating a delayed RFO synthesis when lipid accumulation continued into the maturation phase (Section 2.2, Figure 3). However, the RFO content at maturity did not show a significant difference between the two mutants and the wild-type Williams 82. The sucrose levels at maturity in the two mutants, FN300012 and FN301952, were elevated by 1.7-fold (*p* = 0.021) and 1.8-fold (*p* = 0.017), respectively.

### 3.4. A Delayed Switch towards RFO Accumulation Leads to Extended Oil and Protein Accumulation

**Figure 5:**
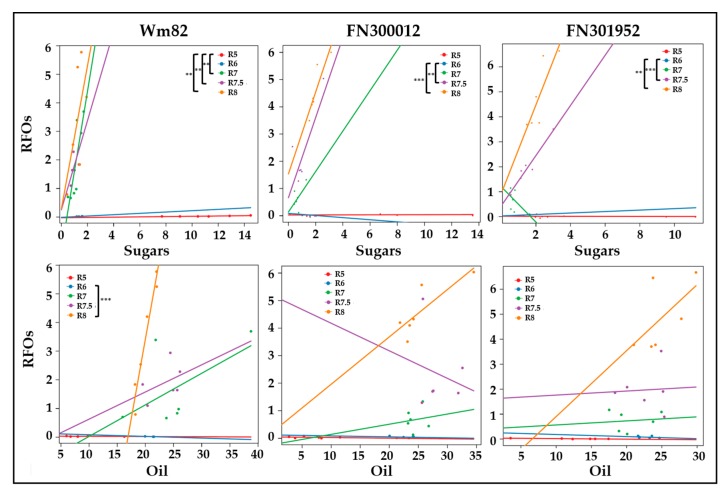
Linear models between sugars and RFOs (top panel) or oil content and RFOs (bottom panel) represented by regression lines, color coded for developmental time points (R5–R8). Statistically significant differences in slopes between time points that represent a change in the direction of carbon flow are marked for each of the three lines, namely Williams 82, FN300012, and FN301952. ** *p* < 0.01 and *** *p* < 0.001. Units for both axes represent % biomass. Sugars represent the sum of glucose, fructose, and sucrose. RFOs represent raffinose and stachyose together.

The change in resource allocation between sugars, oil, and RFOs was investigated using a correlation-based approach followed by linear modeling [46]. Correlation matrices between biomass components including sugars (glucose + fructose + sucrose), RFOs (raffinose + stachyose), oil, and protein, at all stages were generated for Williams 82 and the two related FN lines, FN300012 and FN301952 (Figure S2, Supplementary Materials). A positive correlation between sugars and RFOs was observed in the wild type in all stages of development except maturity, indicating the channeling of free sugars towards RFOs from the beginning of storage reserve deposition. This correlation, however, was found to be negative at R6 in FN300012, and no significant positive correlations were observed until R7.5 in both FN300012 and FN301952. The results suggest that the allocation of carbon from sugars to RFOs is delayed and may contribute to enhanced lipid accumulation. Linear modeling (where the difference in slopes of the regression lines are compared over time) used to identify the relationship between sugars and RFOs revealed a significant difference between R5 and maturation phases (R7 and R8) (Figure 5). This difference in slope, indicated by a significant interaction term (*p*-value <0.05), may represent a metabolic shift. Linear models have been used previously to identify compounds that are differentially directed towards metabolic fates under regulation [46]. In the types of metabolic shifts detected in soybean, the biochemical relationship between classes of compounds changes as a function of developmental time. The difference was not apparent until R7.5 in the mutant lines FN300012 and FN301952, consistent with extended oil accumulation and delayed RFO production.

Out of the possible paired relationships examined using the linear modeling approach (sugars, RFOs, oil, and protein) over time (R5–R8), other significant differences in slopes were identified related to oil and RFO accumulation, indicating that the carbon from the lipid may be repurposed for RFO synthesis in Williams 82 (Figure 5). Although not statistically significant, a positive relationship between oil and RFOs was observed in wild-type Williams 82 at R7, which was not observed until R8 in both the mutant lines as indicated by the slope of regression lines.

### 3.5. Cysteine Content That Contributes to Protein Quality Peaked Earlier in Development

**Figure 6:**
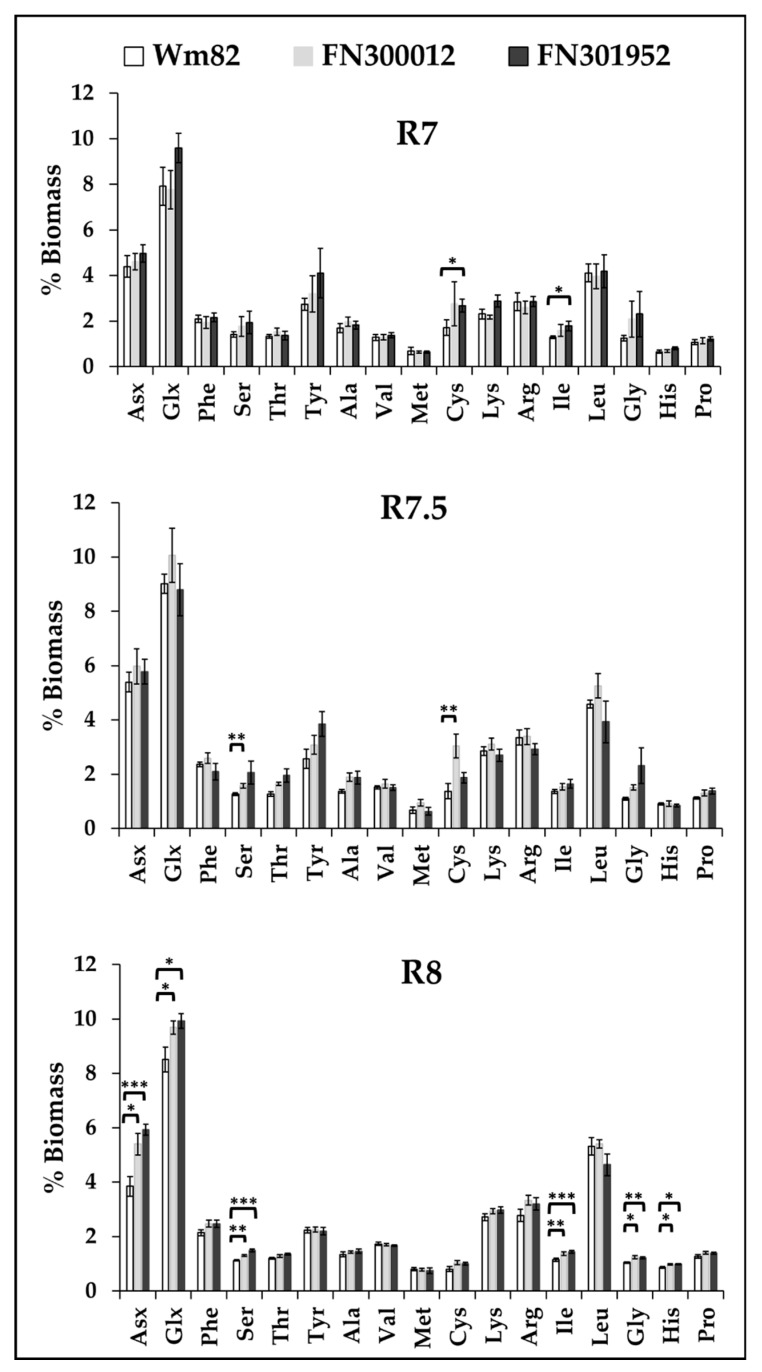
Amino acid composition of the total protein between the three lines, Williams 82, FN300012, and FN301952, at R7 (beginning of maturation), R7.5 (mid-maturation), and R8 (maturity). Values represent a mean (*n* = 6). Student’s *t*-test was used for statistical analyses between the wild type and the two mutants. * *p* < 0.05, ** *p* < 0.01, and *** *p* < 0.001.

The protein quality of the meal from soybeans, in addition to total quantity, contributes to the market value. The mutant lines, FN300012 and FN301952, had protein levels greater (Section 3.2) than the wild-type (Williams 82) and were further evaluated for protein quality through quantification of the amino acids. The sulfur-containing essential amino acids, methionine and cysteine, are generally lower in soybeans than other sources of protein; thus, improving the production of these amino acids has received considerable attention [47,48]. Although the levels of these two amino acids at maturity are not significantly different between the two FN lines compared to Williams 82, the content of cysteine is elevated in R7 in FN300012 and R7.5 in FN301952 before declining to wild-type levels (Figure 6), suggesting that the genes involved in either biosynthesis or degradation of these amino acids are affected. The level of serine, that is the precursor of cysteine biosynthesis, is also significantly higher in FN300012 at R7.5. This may indicate that precursors for cysteine biosynthesis are more available and could contribute to enhanced protein quality. The total protein composition at maturity is different in the two mutants compared to the wild type. Asx (asparagine + aspartate), Glx (glutamine + glutamate), serine, isoleucine, glycine, and histidine are all greater in the mutants compared to the wild type (Figure 6), suggesting an altered protein profile possibly resulting from changes to protease activities as a result of the FN mutations.

## 4. Discussion

Interconnected enzymatic steps result in the flow of carbon through central metabolic pathways to the production of valuable biomass components, including oil, protein, and carbohydrates [23,49]. Conventional breeding and transgenic approaches for improving the quality or quantity of biomass reserves often results in rebalancing between components within the seed [18,48]. Protein and lipid are co-produced during development, including during the seed maturation phase when resources from the maternal plant are scarce. During the maturation phase, the production of one storage component likely results from repartitioning of carbon amongst storage reserves. Attempts at rebalancing protein and oil have given mixed results, with strategies to enhance protein such as heterologous protein expression [50] and suppression of specific seed storage proteins [51] providing small gains in the overall protein content due to concurrent changes within the proteome whereas the expression of the *Arabidopsis thaliana* orphan gene *QUA-QUINE STARCH* (*QQS*) in soybean seeds resulted in 4–10% increase in protein predominantly at the expense of starch [52,53]. Conversely, natural variants of *RAFFINOSE SYNTHASE* responsible for RFO accumulation have decreased RFO content and increased sucrose without further gains in oil or protein accumulation [5]. Thus altering a strategic combination of genes to reshuffle carbon and nitrogen between biomass components can improve the quality of soybeans; but the outcomes remain difficult to predict a priori. We hypothesize that since resources (such as carbon) are finite, the reallocation of carbon to lipid and/or protein instead of RFOs could be important to consider for enhanced seed value at maturity.

FN mutagenized populations have a unique advantage in generating random genomic deletions at multiple loci creating multiple phenotypes [37], and provide a means to select lines that have improved seed composition traits. Genome mapping techniques such as array CGH for locating gene deletions and duplications have expanded the use of FN mutagenized populations as a resource for reverse genetic studies. A number of examples now indicate enhanced protein levels through gene combinations in lines with high oil ([54] and references within) and the use of FN mutagenized populations to identify lines with similar potential [40,41]. In this study, a set of FN lines with high oil, protein, and/or known genetic alterations was selected based on deletions to central carbon metabolic genes responsible for partitioning the maternal nutrient sources into biomass (Table 1). An initial analysis from a subset of these lines with high oil revealed a strong negative correlation (Figure 2) for most lines as has been previously observed [16,17,18,19,20,21]. The two FN lines, FN300012 and FN301952, however, yielded a combined protein and oil quantity roughly 10% (69.5% and 69.1%, respectively) higher compared to the wild-type level (58.9%) at maturity, under greenhouse conditions, avoiding the inverse correlation between oil and protein.

The lines FN300012 and FN301952 were investigated over the course of seed development to assess changes in biomass that result from maternal contribution and temporal changes in metabolism within the seed [22,23,24,55]. The delayed switch in carbon allocation towards raffinose and stachyose occurred concomitantly with prolonged lipid accumulation in FN300012 and FN301952. Furthermore, the decline of lipids generally associated with the maturation period in oilseeds [28,29,30] was delayed in FN300012 and not apparent in FN301952, leading to an increased final oil content. The source of excess carbon deposited into lipids, however, remains unclear since significant decreases in final RFO levels were not observed. The sucrose and protein contents at maturity were also higher in the two mutants, thus the dietary fiber or other unquantified biomass pools could offset the increases in lipids. Indeed, FN300012 was selected due to a knockout in chromosome 1 (~0.7 Mb) that contained a gene coding for a putative UDP-glucose 6-dehydrogenase activity (Glyma01g06970, E.C:1.1.1.22) identified using CGH analysis and catalogued in SoyBase [56]. This enzyme has been shown to enable cell wall polysaccharide biosynthesis as the seed matures [57]. In addition, FN301952 had a ~2.3 Mb homozygous deletion in chromosome 3 that contained three glycolytic enzymes, namely an aldolase (Glyma03g34950), a putative enolase (Glyma03g34830), and a pyruvate kinase (Glyma03g34740), all of which were predicted to be localized in cytosol using WoLF PSORT [58]. The loss of cytosolic components of glycolysis may impact the available hexose phosphates in the chloroplast and result in increased de novo lipid synthesis, which would be distinct from recent strategies to augment lipid levels by reducing lipases in oilseeds, including *Arabidopsis* [59], rapeseed [60], *Jatropha* [61], and soybean [62].

Proteolysis of storage proteins in developing seeds has previously been described [63], and transcripts of several proteases and protein degradation systems are known to be expressed throughout seed development [28,31]. The two FN lines contained several proteases among the list of deleted genes, likely contributing to reduced or delayed proteolysis leading to loss of protein during the maturation phase observed in Williams 82. The mutant lines contained higher protein-linked cysteine content important to meal quality [48], during mid-maturation phase. A gene related to the methionine degradation pathway, S-adenosyl methionine synthetase (Glyma03g34120), that was deleted in FN301952 may affect methionine levels affecting degradation and produce cysteine that accumulates in protein. Several protease-related genes were deleted in the mutant lines and, along with others that impact sulfur-enriched proteins, remain a target for higher quality protein at maturity.

## 5. Conclusions

Fast neutron mutagenesis resulted in lines with different carbon partitioning to storage reserves and served as a valuable resource for identification and characterization of novel phenotypes and their genetic basis. We identified two FN lines with increased oil and protein by a combined 10%. Temporal changes in biomass composition revealed a delayed carbon allocation to RFO synthesis in the mutant lines compared to the wild type. Further studies on the temporal changes in metabolism during soybean development can be used to identify candidate genes for elite soybean germplasm production.

## Figures and Tables

**Table 1:** Fast neutron (FN) mutagenized population used for preliminary oil and protein analysis in 2018.

FN Descriptor	Descriptors Used Here	50-Seed Weight (g)	Genes of Interest within FN Deletion or Phenotype Used as Selection Criteria
Williams 82	WT-Wm 82	10.05	Wild type
FN300660	FN300660	11.87	α/β hydrolase, Glyma14g09181
FN300088	FN300088	10.49	α/β hydrolase, Glyma17g35990
FN300068.01.01.M4	FN300068	13.62	F1,6BPase, Glyma16g28310
FN300012.02.BC1F3	FN300012	11.15	UDP-Gluc-6DH, Glyma01g06970
FN300026.02.01.M4	FN300026	11.57	PEPCK, Glyma04g09510
FN300108	FN300108	7.66	ACX2, Glyma06g43840
FN301952.01.01.M4	FN301952	13.33	Enolase, Glyma03g34830
FN301653.01.01.M4	FN301653	12.11	F1,6BPase, Glyma18g41940
M92-220	WT-M92-220	13.65	Wild type
FN0171815	FN0171815	14.63	high oil
FN0110332.03.M3	FN0110332	11.98	Aldolase, Glyma12g24190
FN0170714.03.01.02.M5	FN0170714	16.3	F1,6BPase Glyma18g41940
P1082Daar541BMN15 ^1^	P1082D	10.76	high protein
FN0175143	FN0175143	13.8	high oil
FN0171855.08.01.01.10.M6	FN0171855	13.32	PEPCK Glyma04g09510
5R16CO1Ddar117MN15 ^1^	5R16CO1D	12.92	high oil
FN0170904.01.M3	FN0170904	13.93	F1,6BPase Glyma16g28310
FN0171734	FN0171734	9.93	SUS1/4 Glyma09g08550
PO559Caar541MN15 ^1^	PO559C	12.75	high protein + oil
FN0141075.03.04.10.M5	FN0141075	12.29	ACX1 Glyma01g41600
1R37C45cbdaar254MN15 ^1^	1R37C45	13.28	high protein + oil
FN0171466	FN0171466	14.23	high oil
FN0173708	FN0173708	13.32	high oil
FN0175116	FN0175116	13.08	high oil
FN0111996	FN0111996	13.53	PXA1 Glyma11g38160
FN0173054.M2	FN0173054	14.18	PGM Glyma08g04890

^1^ Lines belonged to the 2015 core collection with no FN ID.

## Supplementary Materials

The following are available online at https://www.mdpi.com/2218-1989/10/1/18/s1, Figure S1: Total lipid content represented as percent biomass in the selected FN population derived from the wild type, M92-220 (top panel), and the ones derived from Williams 82 (bottom panel). Figure S2: Correlation matrices showing temporal relationships between the four biomass components, namely, sugars, RFOs, oil, and protein in the two FN lines, FN300012 and FN301952, along with the wild-type Williams 82. Grey shaded values represent the biologically relevant comparisons. * *p* < 0.05, ** *p* < 0.01, and *** *p* < 0.001. Table S1: Multiple reaction monitoring (MRM) method settings for the quantification of sugars using SCIEX Triple Quad^TM^ 6500+ system. Table S2: Oil and protein content of the eleven fast neutron mutant lines along with their wild-type backgrounds used for correlation, as described in Figure 2. Table S3: Fresh weights of seeds used for analyses at different stages of seed development along with oil, protein, sucrose, and RFO content that represent the data presented in Figure 3; Figure 4. The R scripts for correlation analysis and the linear modeling as well as the data used are available at https://github.com/victorfrnak/allen_lab.
