# Supplementary material for: On the Inverse Correlation of Protein and Oil: Examining the Effects of Altered Central Carbon Metabolism on Seed Composition Using Soybean Fast Neutron Mutants
Source: Metabolites. 2019 Dec 28;10(1):18. doi: 10.3390/metabo10010018 (PMC7022410; doi:10.3390/metabo10010018)
Supplement: Supplementary file 1 [file metabolites-10-00018-s001.zip › Supplementary tables.docx]

**Table S1: Multiple Reaction Monitoring (MRM) method settings for the quantification of sugars using SCIEX Triple Quad^TM^ 6500+ system**

| **Metabolite** | **Q1** | **Q3** | **Collision Energy** | **Declustering potential** | **Collision cell exit potential** |
| --- | --- | --- | --- | --- | --- |
|  | **Mass** | **Mass** | **(eV)** | **(eV)** | **(eV)** |
| Glucose/Fructose* | 179 | 89 | -10 | -25 | -11 |
| Maltose | 341 | 161 | -10 | -65 | -13 |
| Raffinose | 503 | 179 | -28 | -175 | -11 |
| Ribitol | 151 | 89 | -16 | -50 | -9 |
| Stachyose | 665 | 179 | -38 | -80 | -13 |
| Sucrose/Galactinol* | 341 | 179 | -18 | -110 | -13 |

*These compounds are chromatographically separated

**Table S2: Oil and protein content of the eleven Fast Neutron lines along with their wild type backgrounds used for correlation described in Figure 2**

| **Genotype** | **Replicate** | **Oil (% biomass)*** | **Protein (% biomass)*** |
| --- | --- | --- | --- |
| WT-Wm 82 | A | 16.94 | 45.81 |
| WT-Wm 82 | C | 20.3 | 36.38 |
| FN300660 | A | 21.99 | 40.5 |
| FN300660 | B | 22.71 | 37.44 |
| FN300660 | C | 23.19 | 39.81 |
| FN300660 | D | 22.11 | 41.69 |
| FN300012 | A | 22.79 | 40.13 |
| FN300012 | B | 23.83 | 40.75 |
| FN300012 | C | 21.64 | 39.06 |
| FN300012 | D | 21.7 | 37.75 |
| FN301952 | A | 24.64 | 43.13 |
| FN301952 | B | 27.03 | 30.69 |
| FN301952 | C | 21.64 | 39.75 |
| FN301952 | D | 21.16 | 40.94 |
| WT-M92-220 | C | 18.78 | 46.19 |
| WT-M92-220 | D | 19.22 | 38.63 |
| FN0171855 | A | 21.77 | 40.94 |
| FN0171855 | B | 26.04 | 32 |
| FN0171855 | C | 27.01 | 30.5 |
| 5R16CO1D | A | 26.4 | 27.06 |
| 5R16CO1D | B | 29.94 | 27.63 |
| 5R16CO1D | C | 30.32 | 29.06 |
| 5R16CO1D | D | 27.05 | 30.69 |
| FN0170904 | A | 26.18 | 32.31 |
| FN0170904 | B | 28.34 | 33.13 |
| FN0170904 | C | 23.15 | 36.31 |
| FN0170904 | D | 23.36 | 33.88 |
| FN0171734 | A | 26.28 | 30.13 |
| FN0171734 | B | 24.76 | 29.19 |
| FN0171734 | C | 27.62 | 29.63 |
| FN0171734 | D | 26.68 | 29.44 |
| FN0171466 | A | 24.05 | 34.56 |
| FN0171466 | B | 25.54 | 29.25 |
| FN0171466 | C | 24.89 | 29.5 |
| FN0173708 | A | 24.81 | 30.25 |
| FN0173708 | B | 26.49 | 29.88 |
| FN0173708 | C | 23.5 | 32.25 |
| FN0175116 | A | 23.34 | 34.81 |
| FN0175116 | B | 22.35 | 30.81 |
| FN0175116 | C | 25.63 | 31.06 |
| FN0175116 | D | 25.42 | 36.44 |
| FN0173054 | A | 22.05 | 37.81 |
| FN0173054 | B | 22.68 | 38.31 |
| FN0173054 | C | 25.41 | 28.31 |
| FN0173054 | D | 25 | 30.94 |

*Units represent mg per 100 mg tissue represented as % biomass.

**Table S3: Fresh weights of seeds used for analyses at different stages of seed development along with oil, protein, sucrose and RFO content that represent the data presented in Figures 3 and 4.**

|  | **Stage** | **Fresh weight (mg seed^-1^)** | **Oil*** | **Protein*** | **Sucrose*** | **Raffinose*** | **Stachyose*** |
| --- | --- | --- | --- | --- | --- | --- | --- |
| **Wm82** | R5 | 57.4 ± 3.69 | 9.13 ± 1.52 | 34.92 ± 1.39 | 7.56 ± 0.77 | 0.02 ± 0.003 | 0 ± 0 |
|  | R6 | 294.65 ± 24.1 | 20.93 ± 0.36 | 36.04 ± 3.24 | 1.35 ± 0.08 | 0.01 ± 0.001 | 0 ± 0 |
|  | R7 | 254.72 ± 27.41 | 25.3 ± 3.07 | 38.83 ± 2.25 | 1.03 ± 0.17 | 0.53 ± 0.18 | 1.15 ± 0.39 |
|  | R7.5 | 203.67 ± 10.24 | 23.51 ± 1.16 | 42.1 ± 1.88 | 1.05 ± 0.12 | 0.49 ± 0.05 | 1.13 ± 0.19 |
|  | R8 | 135.72 ± 12.11 | 19.98 ± 0.71 | 38.92 ± 1.28 | 1.22 ± 0.21 | 0.71 ± 0.15 | 2.33 ± 0.6 |
| **FN300012** | R5 | 43.25 ± 4.73 | 6.61 ± 1.67 | 37.71 ± 1.31 | 3.36 ± 0.96 | 0.01 ± 0.004 | 0 ± 0 |
|  | R6 | 284.57 ± 7.55 | 22.57 ± 0.67 | 40.13 ± 2.66 | 1.17 ± 0.27 | 0.03 ± 0.01 | 0 ± 0 |
|  | R7 | 290.08 ± 20.95 | 24.47 ± 0.6 | 41.72 ± 2.89 | 0.71 ± 0.13 | 0.21 ± 0.04 | 0.42 ± 0.12 |
|  | R7.5 | 240.36 ± 4.16 | 28.48 ± 1.23 | 49.47 ± 3.25 | 1.12 ± 0.31 | 0.38 ± 0.09 | 1.47 ± 0.36 |
|  | R8 | 181.67 ± 9.49 | 25.46 ± 1.88 | 44.05 ± 0.82 | 2.03 ± 0.23 | 0.88 ± 0.09 | 3.01 ± 0.29 |
| **FN301952** | R5 | 37.1 ± 2.25 | 12.41 ± 2.02 | 38.85 ± 1.94 | 2.97 ± 0.62 | 0.01 ± 0.004 | 0 ± 0 |
|  | R6 | 332.95 ± 12.22 | 22.79 ± 0.5 | 40.93 ± 1.31 | 1.58 ± 0.27 | 0.07 ± 0.01 | 0 ± 0.002 |
|  | R7 | 251.9 ± 10.8 | 20.74 ± 1.2 | 46.72 ± 2 | 0.64 ± 0.05 | 0.21 ± 0.04 | 0.51 ± 0.13 |
|  | R7.5 | 230.64 ± 21.1 | 22.72 ± 1.21 | 46.18 ± 1.23 | 1.5 ± 0.34 | 0.42 ± 0.1 | 1.16 ± 0.19 |
|  | R8 | 180.1 ± 7.74 | 25 ± 1.31 | 44.1 ± 1.08 | 2.19 ± 0.26 | 0.94 ± 0.14 | 3.06 ± 0.39 |

*Values represent % biomass (or mg per 100 mg tissue) ± standard error of mean (*n* = 6)
