# Supplementary figures and images for: On the Inverse Correlation of Protein and Oil: Examining the Effects of Altered Central Carbon Metabolism on Seed Composition Using Soybean Fast Neutron Mutants
Source: Metabolites. 2019 Dec 28;10(1):18. doi: 10.3390/metabo10010018 (PMC7022410; doi:10.3390/metabo10010018)

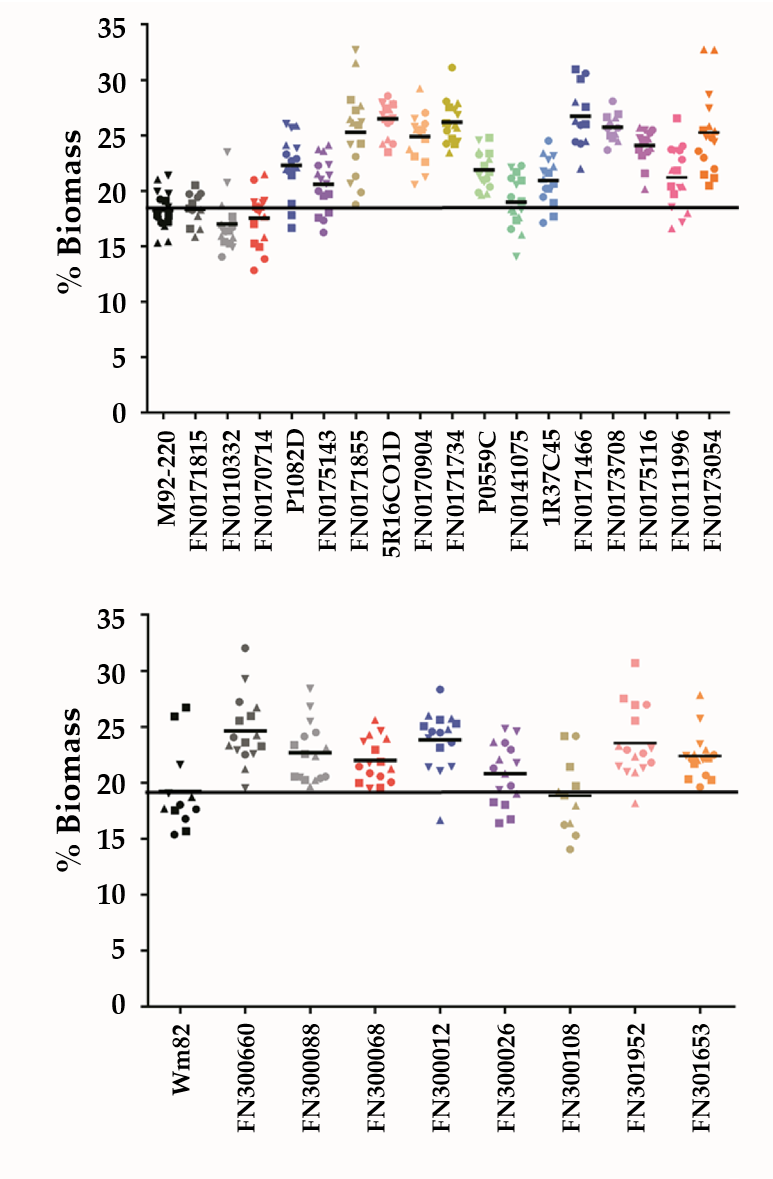

Supplement: Supplementary file 1 [file metabolites-10-00018-s001.zip › Figure S1.tif]

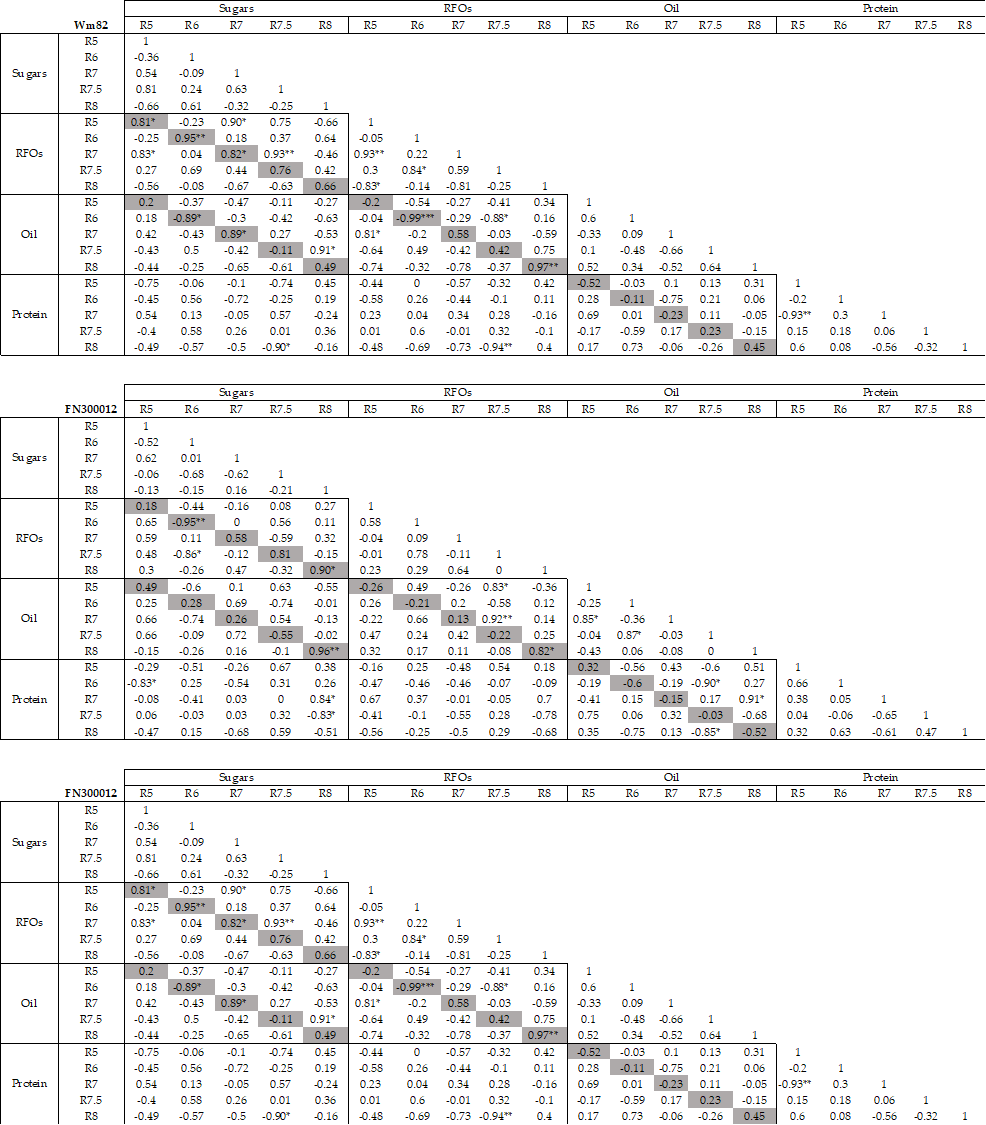

Supplement: Supplementary file 1 [file metabolites-10-00018-s001.zip › Figure S2.tif]
